# Global, regional, and national burdens of pancreatitis in children and adolescents aged 0–24 years from 1992 to 2021: a trend analysis based on the global burden of disease study 2021

**DOI:** 10.3389/fpubh.2025.1527569

**Published:** 2025-06-26

**Authors:** Ying-han Deng, Huabin Qiu, Kangming Huang, Yanbin Huang, Fuming Lian, Yun Chen, Hongbin Chen

**Affiliations:** ^1^Department of Gastroenterology, Sanming First Hospital Affiliated to Fujian Medical University, Sanming, China; ^2^Department of Orthopedics, The Second Affiliated Hospital of Fujian Medical University, Quanzhou, China

**Keywords:** pancreatitis, burden, children, adolescents, acute pancreatitis, chronic pancreatitis

## Abstract

**Background:**

The younger onset of pancreatitis presents a significant public health challenge. This study aims to analyze the global burden of pancreatitis in younger populations based on the Global Burden of Disease 2021.

**Methods:**

This study uses incidence rates and Disability-Adjusted Life Years (DALYs) to assess the burden of pancreatitis. Joinpoint modeling was used to assess the trend of the burden. Age-Period-Cohort (APC) modeling was used to assess the annual percentage changes by age, as well as the period and cohort relative risks. Norpred modeling was used to predict the burden through 2040.

**Results:**

In 2021, the global incidence of pancreatitis among younger individuals was 9.16/100,000 (95% UI 5.74–13.85), with an annual average percentage change (AAPC) of 0.13 (95% CI 0.12–0.14). The DALYs was 6.36/100,000 (95% UI 5.21–7.97), with an AAPC of −0.93 (95% CI -1.01 to −0.85). Global incidence rates of pancreatitis have notably increased since 1999, while the overall burden of DALYs has decreased over the past 30 years. In the APC model, different age groups experienced varying risks. According to the Norpred predictive model, by 2040, the global incidence of pancreatitis among younger individuals is projected to reach 313,567 cases, with an incidence rate of 9.07/100,000.

**Conclusion:**

Globally, the incidence of pancreatitis in younger individuals has increased over the past three decades. Urgent policy interventions are needed to address healthcare inequities and alleviate this burden.

## 1 Introduction

Pancreatitis represents a formidable clinical and public health challenge owing to its multifactorial pathogenesis, heterogeneous clinical manifestations encompassing local and systemic complications, and propensity for recurrent exacerbations and progression to chronic disease. The incidence of acute pancreatitis has been increasing with societal development (1). Emerging evidence indicates that individuals who experience an episode of acute pancreatitis are at an elevated risk of progressing to chronic pancreatitis (2), and the incidence of chronic pancreatitis is also rising (3). As a result, the overall burden of pancreatitis has increased over time. Notably, clinical observations suggest a growing trend of pancreatitis among younger individuals, including adolescents, young adults, and children, with pediatric pancreatitis imposing a greater burden than previously recognized (4).

However, most epidemiological studies on pancreatitis rely on local data, which may introduce bias and limit generalizability. The Global Burden of Disease (GBD) study, utilizing extensive data sources and advanced statistical modeling techniques, provides a robust framework for comprehensively assessing the burden of pancreatitis. In this analysis, we examined data from 1992 to 2021, focusing on individuals aged 0–24 years. Global trends were stratified by age, sex, and the Socio-Demographic Index (SDI), with findings reported at both the regional and national levels.

## 2 Methods

### 2.1 Study population and data collection

The GBD database^1^ is one of the most comprehensive and systematic epidemiological studies conducted globally. GBD 2021 provides a comprehensive evaluation of 371 diseases and injuries, along with 88 risk factors worldwide (5). For this analysis, we obtained data on pancreatitis among individuals aged 0–24 years from the GBD 2021 database, covering the period from 1992 to 2021 across 204 countries, 21 regions, and 5 SDI regions. Our primary focus was on incidence rates and Disability-Adjusted Life Years (DALYs) to assess the epidemiological trends in pancreatitis.

The 0–24 age group was divided into five subgroups: 0–4 years, 5–9 years, 10–14 years, 15–19 years, and 20–24 years. We extracted the corresponding data for these subgroups from the GBD database and recalculated both all-age and age-standardized rates for the 0–24 age group. According to the World Health Organization (WHO), children are defined as individuals aged 0–9 years, and adolescents as those aged 10–19 years (6, 7). Accordingly, we categorized the five subgroups as early childhood (0–4 years), late childhood (5–9 years), adolescence (10–14 years), older adolescence (15–19 years), and young adulthood (20–24 years). This detailed stratification aims to better capture the developmental processes within this age range and enhance public understanding of these key life stages.

### 2.2 Statistical analysis

#### 2.2.1 Joinpoint regression model analysis

This study employed a joinpoint regression model based on the Poisson distribution to examine the changing trends in the burden of pancreatitis over the past 30 years among individuals aged 0–24 years. Joinpoint analysis was used to quantify these trends, with the annual percentage change (APC) reported along with the identified joinpoints. Long-term trends were expressed as the average annual percentage change (AAPC). A two-sided *p* < 0.05 was considered statistically significant, indicating a rising or declining trend. If the result was not statistically significant, the trend was considered stable.

#### 2.2.2 Age-period-cohort (APC) model

This study employed the Age-Period-Cohort (APC) model framework to explore potential trends in the incidence and DALYs associated with pancreatitis across age, period, and birth cohort dimensions (8). The APC model aims to disentangle the contributions of biological aging processes, along with technological and social factors, to observed disease trends (9). It typically fits a log-linear Poisson model to observed rates on a Lexis diagram, capturing the cumulative effects of age, period, and cohort.

To address the inherent linear dependency among age, period, and cohort (birth cohort = period – age), we generated estimable APC parameters and functions without imposing arbitrary constraints on model parameters. The fitted APC model estimated the overall time trend in rates, expressed as the annual percentage change (net drift, % per year). Additionally, it estimated time trends for each age subgroup, referred to as local drifts, which represent the annual percentage change within the age-specific rates.

The outputs of the APC model included fitted longitudinal age-specific rates for a reference cohort, adjusted for period deviations, to reflect the natural history associated with aging. For the period and cohort dimensions, relative risks were calculated as age-specific rate ratios for each period or cohort, compared with a chosen reference period or cohort. Both period and cohort rate ratio curves encompassed the full range of net drift values (10). The selection of the reference period or cohort was arbitrary but essential for comparative interpretation.

#### 2.2.3 Norpred

We employed the Norpred model, a log-linear Age-Period-Cohort (APC) model, to forecast global incidence rates and DALYs for pancreatitis, disaggregated by region, gender, and age group, through 2040. For the most recent three to four 5-year observation periods, a power function was utilized to extrapolate and refine growth trends. The linear trend observed over the past decade was projected to decline by 25, 50, and 75% during the second, third, and fourth forecast periods, respectively (11).

The estimated number of new cases in 2040 was derived by calculating a weighted average of the predicted incidence rates from the final two forecast periods. These rates were then applied to population projections provided by the United Nations for each country (12).

## 3 Results

### 3.1 Global burden of pancreatitis in 0–24 years in 2021

In 2021, the global age-standardized incidence rate (ASIR) of pancreatitis among individuals aged 0–24 years was 9.16 per 100,000 population (95% UI: 5.74–13.85), while the ASR for DALYs was 6.36 (95% UI: 5.21–7.97). Among the five age groups, the 20–24 age group exhibited the highest incidence rate, at 18.54 (95% UI: 12.14–27.26), and the highest DALYs, at 19.23 (95% UI: 16.18–23.80). In contrast, the 0–4 age group had the lowest incidence rate, at 2.24 (95% UI: 1.37–3.56), and the lowest DALYs, at 0.76 (95% UI: 0.47–1.14) (Table 1 and Supplementary material 1).

**Table 1:** Incidence and annual average percentage changes (AAPCs) for pancreatitis among individuals aged 0–24 years in global, 21 GBD regions and 5 SDI regions from 1992 to 2021.

Location	1992		2021		1992–2021
	Number (95% UI)	ASDR (95% UI)	Number (95% UI)	ASDR (95% UI)	AAPC (95% UI)
Global	248013.0 (152546.6–381712.7)	8.83 (5.43–13.59)	305352.3 (191619.2–461132.7)	9.16 (5.74–13.85)	0.13 (0.12–0.14)
Regions					
Andean Latin America	2568.7 (1598.6–3893.9)	11.39 (7.10–17.23)	3370.1 (2272.4–4878.1)	10.85 (7.29–15.75)	−0.16 (−0.23 to −0.09)
Australasia	755.0 (455.5–1169.5)	8.66 (5.19–13.49)	861.6 (526.5–1323.6)	8.37 (5.09–12.90)	−0.12 (−0.14 to −0.09)
Caribbean	1606.2 (953–2533.5)	8.48 (5.02–13.39)	1658.3 (983.2–2585.1)	8.33 (4.93–13.02)	−0.06 (−0.1 to −0.02)
Central Asia	4156.1 (2524.1–6356.1)	11.32 (6.89–17.28)	4626.9 (2832.7–7042.6)	11.44 (7.01–17.39)	0.03 (0–0.07)
Central Europe	4859.0 (3108.5–7148.2)	9.54 (6.09–14.08)	2504.8 (1791.4–3353.5)	7.94 (5.65–10.66)	−0.63 (−0.67 to −0.59)
Central Latin America	11600.1 (7286.4–17478.2)	11.62 (7.3–17.51)	13631.0 (8704.6–20229.4)	12.11 (7.73–18.02)	0.15 (0.14–0.15)
Central Sub-Saharan Africa	1880.5 (1062.9–3056.6)	5.50 (3.13–8.89)	4426.1 (2484.3–7197.3)	5.44 (3.07–8.82)	−0.04 (−0.05 to −0.02)
East Asia	46965.0 (26613.6–76004.9)	7.43 (4.18–12.10)	19927.5 (11556.4–31,892)	4.64 (2.69–7.41)	−1.68 (−1.83 to −1.53)
Eastern Europe	19294.7 (11753.5–30097.6)	22.81 (13.90–35.56)	13618.8 (8363.1–20962.6)	23.74 (14.59–36.54)	0.14 (0.08–0.18)
Eastern Sub-Saharan Africa	7245.6 (4159.9–11619.3)	5.92 (3.42–9.44)	15261.0 (8838.8–24406.7)	5.87 (3.41–9.36)	−0.03 (−0.06 to −0.01)
High-income Asia-Pacific	8762.3 (5486.3–13182.1)	12.98 (8.10–19.62)	5206.9 (3422.5–7436.8)	12.05 (7.91–17.28)	−0.27 (−0.3 to −0.24)
High-income North America	13422.5 (8712.5–19,216)	11.85 (7.65–17.03)	14489.5 (11122.1–18237.8)	11.38 (8.69–14.37)	−0.15 (−0.17 to −0.12)
North Africa and Middle East	14840.6 (8880.9–22932.3)	7.12 (4.28–10.98)	20211.5 (12152–31060.2)	7.00 (4.21–10.75)	−0.06 (−0.08 to −0.04)
Oceania	195.7 (108.8–319.4)	4.93 (2.75–8.02)	351.3 (197.7–567.1)	4.77 (2.69–7.68)	−0.11 (−0.12 to −0.11)
South Asia	67775.1 (40787.6–105648.2)	10.77 (6.49–16.74)	128025.6 (77681.3–197945.7)	13.92 (8.42–21.60)	0.91 (0.87–0.96)
Southeast Asia	16979.4 (9883.7–27,155)	6.30 (3.67–10.08)	18864.9 (11187.1–29480.1)	6.27 (3.71–9.83)	−0.01 (−0.02 to −0.01)
Southern Latin America	2356.4 (1522.1–3436.5)	9.80 (6.33–14.30)	2956.1 (1894.5–4297.5)	10.88 (6.95–15.87)	0.37 (0.32–0.43)
Southern Sub-Saharan Africa	2100.1 (1235.7–3303.4)	6.57 (3.88–10.33)	2444.2 (1437.9–3835.6)	6.34 (3.73–9.96)	−0.13 (−0.18 to −0.08)
Tropical Latin America	3048.2 (1950.8–4460.4)	3.55 (2.27–5.19)	2812.2 (1783.8–4121.7)	3.07 (1.93–4.52)	−0.49 (−0.58 to −0.41)
Western Europe	9345.3 (6175.6)	6.57 (4.27–9.68)	9039.9 (6414.5–12373.8)	7.10 (5.01–9.78)	0.26 (0.2–0.32)
Western Sub-Saharan Africa	8256.5 (4820–13128.7)	6.96 (4.09–11.01)	21064.0 (12315.1–33405.6)	7.17 (4.21–11.33)	0.1 (0.05–0.14)
Socio-demographic index regions					
High SDI	34921.5 (22487.6–51500.4)	10.04 (6.42–14.88)	30775.8 (22242.1–40,964)	9.35 (6.71–12.52)	−0.25 (−0.28 to −0.23)
High-middle SDI	50262.7 (31816.8–74584.7)	10.15 (6.40–15.11)	35750.2 (23283–51,535)	9.00 (5.85–13.00)	−0.42 (−0.47 to −0.38)
Middle SDI	78196.3 (46509.9–122027.1)	8.01 (4.76–12.52)	82259.2 (50898.9–125292.3)	8.47 (5.23–12.92)	0.23 (0.21–0.26)
Low-middle SDI	61790.7 (37012.9–96904.4)	9.09 (5.46–14.21)	103918.7 (62623–161447.2)	10.67 (6.42–16.61)	0.57 (0.54–0.59)
Low SDI	22647.1 (13341.8–35846.2)	7.43 (4.40–11.69)	52473.0 (31288.5–82611.2)	7.93 (4.74–12.46)	0.2 (0.17–0.22)

ASIR, age-standardized incidence rate; AAPC, average annual percentage change.

At the regional level, Eastern Europe had the highest age-standardized incidence rate (ASIR) for pancreatitis in the 0–24 age group, at 23.74 (95% UI: 14.59–38.54), while Latin America recorded the lowest ASR, at 3.07 (95% UI: 1.93–4.51). Eastern Europe also led in DALYs, with an ASR of 18.03 (95% UI: 13.72–24.08), whereas Australasia had the lowest, at 1.28 (95% UI: 0.94–1.79). In terms of case numbers, South Asia reported the highest burden, with 128,025.6 cases (95% UI: 77,681.9–197,945.7), while Oceania had the lowest, with 351.3 cases (95% UI: 197.7–567.1) (Tables 1, 2).

**Table 2:** Disability-adjusted life years (DALYs) and annual average percentage changes (AAPCs) for pancreatitis among individuals aged 0–24 years in global, 21 GBD regions and 5 SDI regions from 1992 to 2021.

Location	1992		2021		1992–2021
	Number (95% UI)	ASDR (95% UI)	Number (95% UI)	ASDR (95% UI)	AAPC (95% UI)
Global	230625.9 (187334.8–301892.7)	8.16 (6.62–10.69)	214847.2 (176261.2–268827.3)	6.36 (5.21–7.97)	−0.93 (−1.01to −0.85)
Regions					
Andean Latin America	6698.1 (4789.2–9137.2)	29.61 (21.25–40.26)	4320.8 (3113.9–5913.9)	13.84 (9.96–18.96)	−2.67 (−3.18 to −2.16)
Australasia	159.9 (126.9–203.1)	1.83 (1.45–2.34)	133.3 (97.9–185.1)	1.28 (0.94–1.79)	−1.15 (−1.95 to −0.35)
Caribbean	1446.5 (1096.8–1943.8)	7.48 (5.65–10.10)	1331.5 (921.1–1917.2)	3.20 (2.76–3.67)	−0.51 (−0.94 to −0.07)
Central Asia	3448.8 (2382.6–4719.8)	9.52 (6.61–12.97)	2891.3 (2213.1–3958.8)	7.25 (5.57–9.89)	−0.99 (−1.38 to −0.6)
Central Europe	4785.9 (4292.1–5439.8)	9.2 (8.23–10.48)	2040.3 (1790.6–2342.6)	6.18 (5.41–7.13)	−1.45 (−2.2 to −0.69)
Central Latin America	13681.0 (12544.8–14839.3)	13.87 (12.73–15.03)	14804.6 (13211.1–16472.8)	12.53 (11.16–13.97)	−0.46* (−1.24–0.32)
Central Sub-Saharan Africa	1809.7 (1035.8–3223.1)	5.75 (3.32–10.19)	4023.1 (2424.2–6,685)	5.31 (3.20–8.85)	−0.28 (−0.45 to −0.11)
East Asia	35737.0 (25601.3–46,182)	5.47 (3.90–7.15)	10843.5 (8191.9–14279.9)	2.5 (1.89–3.30)	−2.72 (−3.11 to −2.33)
Eastern Europe	15444.8 (12327.1–20,365)	17.95 (14.30–23.72)	10314.8 (7840.5–13808.6)	18.03 (13.72–24.08)	−0.04* (−0.87–0.79)
Eastern Sub-Saharan Africa	4772.1 (3001.8–8010.9)	4.17 (2.67–6.85)	10804.0 (6802–16027.9)	4.31 (2.71–6.4)	0.11 (0.05–0.16)
High-income Asia-Pacific	3258.4 (2506.6–4321.1)	4.61 (3.53–6.16)	1166.7 (873.8–1617.1)	2.58 (1.91–3.61)	−1.95 (−2.1 to −1.8)
High-income North America	4330.2 (3825.6–5111.5)	3.86 (3.41–4.55)	4653.1 (4111.1–5479.2)	3.60 (3.17–4.26)	−0.22* (−0.83–0.4)
North Africa and Middle East	6026.2 (4053.4–8793.4)	2.90 (1.96–4.22)	6950.3 (4796–9793.5)	2.41 (1.66–3.39)	−0.65 (−0.83 to −0.47)
Oceania	363.9 (431.0–626.2)	9.46 (3.84–17.73)	498.4 (262.1–858.1)	6.90 (3.63–11.86)	−1.04 (−1.41 to −0.67)
South Asia	90,631 (65481.4–135767.6)	14.96 (10.85–22.33)	92510.2 (67366.4–122,446)	9.69 (7.05–12.85)	−1.47 (−1.9 to −1.03)
Southeast Asia	16087.0 (11283.4–24591.1)	6.02 (4.22–9.20)	15598.7 (11353.6–24103.7)	4.99 (3.63–7.72)	−0.67 (−0.84 to −0.5)
Southern Latin America	2201.8 (1912.5–2520.6)	9.14 (7.94–10.47)	1855.0 (1586.2–2182.2)	6.59 (5.62–7.77)	−1.18 (−1.61 to −0.75)
Southern Sub-Saharan Africa	1108.6 (676.4–1689.4)	3.57 (2.18–5.44)	1600.3 (1050.6–2269.4)	4.13 (2.71–5.87)	0.52 (0.34–0.7)
Tropical Latin America	7343.8 (6659–8222.1)	8.61 (7.80–9.65)	7732.0 (7035.5–8409.9)	8.11 (7.37–8.85)	−0.03* (−0.61–0.56)
Western Europe	4492.8 (4113.9–5000.5)	3.04 (2.78–3.40)	2283.0 (1968.5–2688.5)	1.75 (1.50–2.07)	−1.91 (−2.39 to −1.42)
Western Sub-Saharan Africa	6798.5 (4387.5–10397.3)	6.05 (4.02–8.97)	18492.4 (11985.3–26,024)	6.61 (4.33–9.19)	0.3 (0.15–0.45)
Socio-demographic index regions					
High SDI	14437.2 (12675.6–17076.3)	4.07 (3.56–4.83)	9837.0 (8522.1–11822.4)	2.89 (2.49–3.50)	−1.14 (−1.4 to −0.88)
High-middle SDI	38499.3 (32568.6–47121.1)	7.44 (6.25–9.19)	21710.4 (18038.7–26901.6)	5.3 (4.39–6.59)	−1.25 (−1.77 to −0.72)
Middle SDI	70820.5 (57144.9–87229.8)	7.13 (5.74–8.80)	56079.2 (47182.1–67083.9)	5.65 (4.75–6.76)	−0.81 (−0.92 to −0.7)
Low-middle SDI	81269.7 (59536.1–117823.3)	12.43 (9.15–17.95)	85651.3 (64288.7–112876.9)	8.64 (6.48–11.39)	−1.26 (−1.48 to −1.04)
Low SDI	25413.0 (17341.8–40170.9)	8.90 (6.19–13.77)	41432.1 (31173.5–57002.6)	6.48 (4.89–8.90)	−1.12 (−1.3 to −0.94)

ASDR, age-standardized DALYs rate; AAPC, average annual percentage change. “*”: not statistically significant, *p* > 0.05.

At the national level, the Russian Federation had the highest age-standardized incidence rate (ASIR) for pancreatitis in the 0–24 age group, at 24.7 (95% UI: 15.13–38.22), followed by Ukraine, the Republic of Moldova, Belarus, and Lithuania. For DALYs, the Republic of Kiribati recorded the highest ASR, at 27.16 (95% UI: 14.37–47.70), followed by Tokelau, Guatemala, Niue, and the Republic of Moldova. India reported the largest absolute number of cases, with 128,025.6 (95% UI: 77,681.3–197,945.7) (country-level data provided in Supplementary material 3).

**Figure 1:**
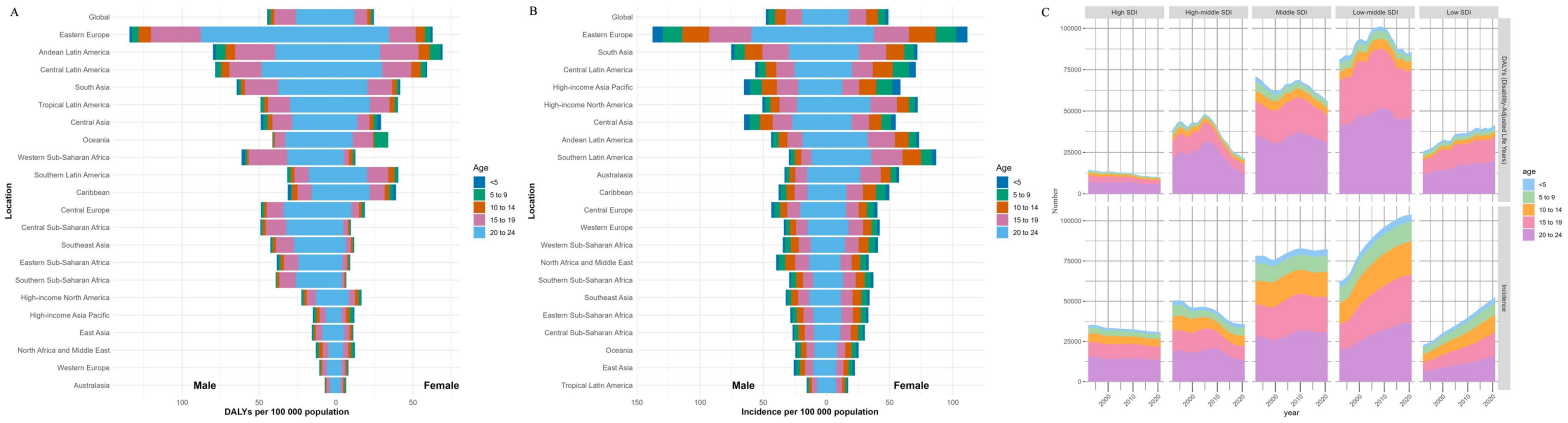
Global, regional incidence and Disability-adjusted life-years (DALYs) of pancreatitis in various age subgroups. **(A)** Disability-adjusted life-years (DALYs) rate for pancreatitis in various age subgroups in Global and 21 GBD regions; **(B)** incidence for pancreatitis in various age subgroups in Global and 21 GBD regions; **(C)** disability-adjusted life-years (DALYs) and incidence cases for pancreatitis in 5 SDI regions.

Globally, over the past 30 years, both the incidence rate and DALYs remained consistently lowest in the 0–4 age group and highest in the 20–24 age group, demonstrating a progressive trend across age groups (Figure 1).

### 3.2 Global trends in the burden of pancreatitis in persons aged 0–24 years over the period 1992–2021

The global age-standardized incidence of pancreatitis among individuals aged 0–24 years exhibited an average annual percentage change (AAPC) of 0.13 (95% CI: 0.12–0.14). In contrast, the AAPC for DALYs showed a declining trend of −0.93 (95% CI: −1.01 to −0.85). Among the five age subgroups, the incidence increased most rapidly in the 15–19 years group, with an AAPC of 0.29 (95% CI: 0.28–0.31), while the 0–4 years group experienced the largest decline in incidence, with an AAPC of −0.23 (95% CI: −0.27 to −0.19). Over the past 30 years, DALYs decreased across all age groups, with the 0–4 years group showing the most substantial decline, at an AAPC of −2.48 (95% CI: −2.6 to −2.36) (Supplementary material 2).

At the regional level, the fastest increase in pancreatitis incidence among individuals aged 0–24 years was observed in South Asia, with an AAPC of 0.91 (95% CI: 0.87–0.96). The steepest decline was recorded in East Asia, with an AAPC of −1.68 (95% CI: −1.83 to −1.53). DALYs for pancreatitis decreased in most regions, although three regions reported increases. The most significant rise occurred in Sub-Saharan Africa, with an AAPC of 0.52 (95% CI: 0.34–0.7), while East Asia exhibited the largest decline in DALYs, with an AAPC of −2.72 (95% CI: −3.11 to −2.33). Regional data are summarized in Tables 1, 2.

**Figure 2:**
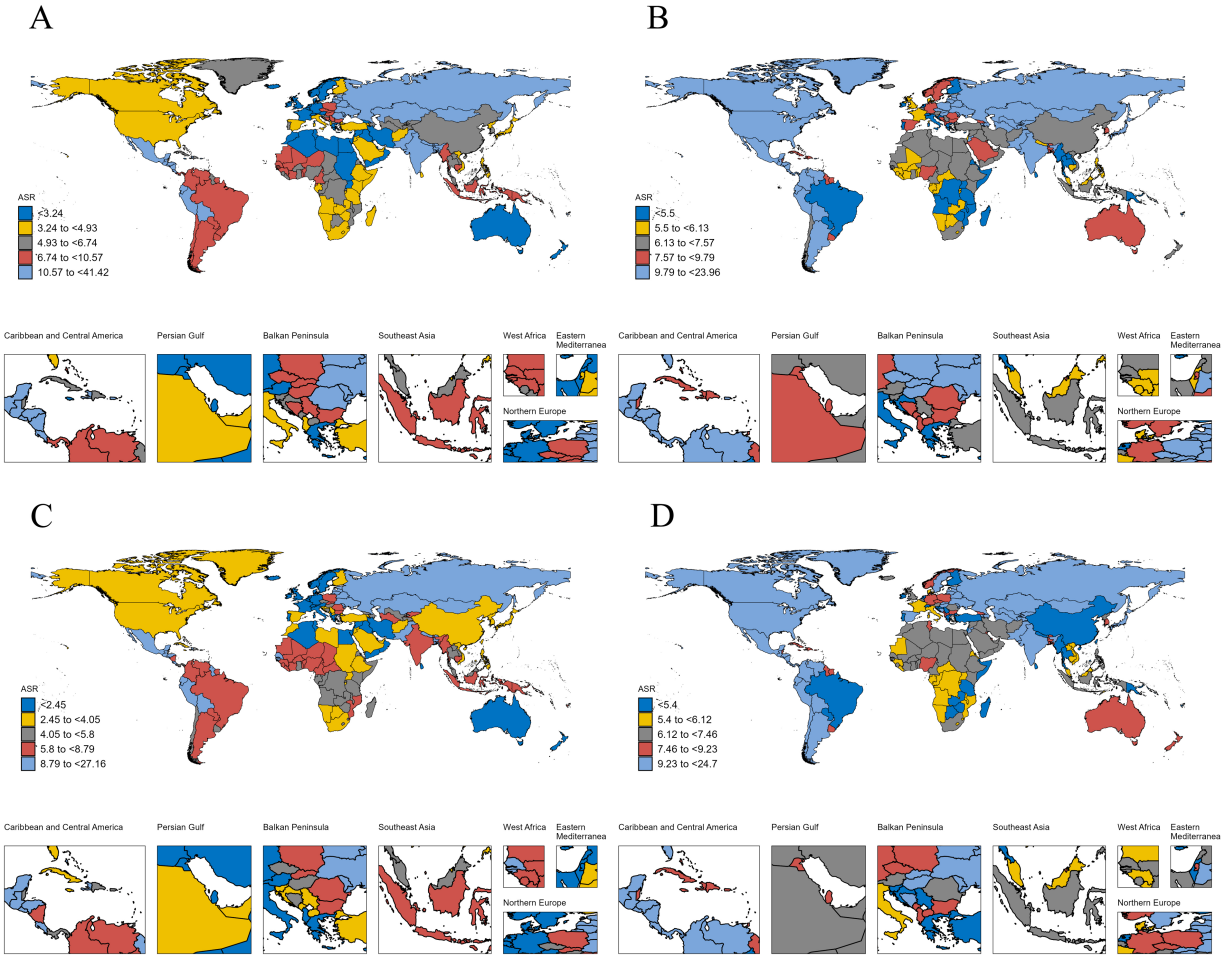
Age-standardized incidence and disability-adjusted life-years (DALYs) rates for pancreatitis among individuals aged 0–24 years across 204 countries worldwide in 1992 and 2021. **(A)** Age-standardized disability-adjusted life-years (DALYs) rate for pancreatitis among individuals aged 0–24 years across 204 countries worldwide in 1992; **(B)** age-standardized disability-adjusted life-years (DALYs) rate for pancreatitis among individuals aged 0–24 years across 204 countries worldwide in 2021; **(C)** age-standardized incidence rate for pancreatitis among individuals aged 0–24 years across 204 countries worldwide in 1992; **(D)** age-standardized incidence rate for pancreatitis among individuals aged 0–24 years across 204 countries worldwide in 2021.

At the national level, Chile reported the fastest increase in pancreatitis incidence among individuals aged 0–24 years, with an AAPC of 1.7 (95% CI: 1.66–1.74), followed by Spain, India, the Netherlands, and New Zealand. The sharpest decline was noted in China, with an AAPC of −1.75 (95% CI: −1.88 to −1.61), followed by Poland, Slovenia, Nepal, and Cyprus. For DALYs, most countries demonstrated a decreasing trend, although 45 out of 204 countries showed an increase. The largest rise in DALYs was observed in Guyana, with an AAPC of 3.01 (95% CI: 2.12–3.9), followed by Tokelau, Niue, and Georgia. Conversely, Luxembourg exhibited the steepest decline, with an AAPC of −3.49 (95% CI: −4.11 to −2.87), followed by Ecuador, Slovenia, Portugal, and Puerto Rico. National-level data are depicted in Figure 2 and Supplementary 4.

**Figure 3:**
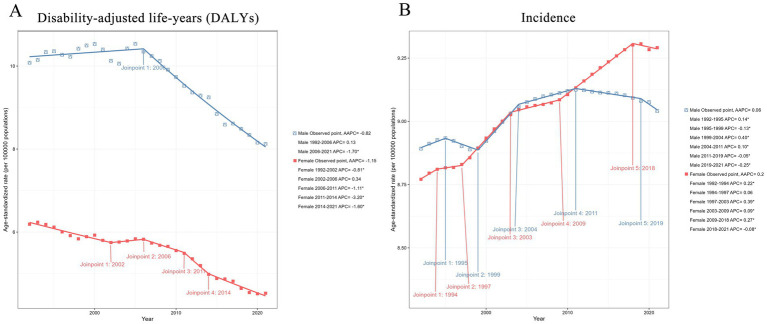
Joinpoint modeling of the age-standardized incidence and disability-adjusted life-years (DALYs) rates of pancreatitis in individuals aged 0–24 years globally. **(A)** Joinpoint modeling of the age-standardized Disability-adjusted life-years (DALYs) rate of pancreatitis in individuals aged 0–24 years globally; **(B)** joinpoint modeling of the age-standardized incidence rate of pancreatitis in individuals aged 0–24 years globally.

In the joinpoint regression model, the global incidence of pancreatitis among individuals aged 0–24 years demonstrated an overall upward trend, with a particularly notable increase observed between 1997 and 2004, followed by a gradual slowdown. Regarding sex differences, the incidence rate was higher in males than in females before 1999. From 1999 to 2011, there was no significant difference in incidence rates between genders. However, after 2011, the incidence among females began to rise while it declined among males, resulting in a significant gender disparity. Meanwhile, the global DALYs burden of pancreatitis among individuals aged 0–24 years showed an overall downward trend throughout the 30-year period. Males consistently exhibited higher DALYs than females across all five SDI regions, with no significant change in the gender gap (Figure 3).

The incidence rates also varied across the 5 SDI regions. In the High SDI region, the incidence of pancreatitis declined from 1995 onward, although the rate of decline slowed, with a slight upward trend emerging after 2015. Throughout the 30-year period, females consistently exhibited higher incidence rates than males, with no significant change in the gender gap. In the High-Middle SDI region, the incidence fluctuated after 1995, but the overall trend was downward. However, the decline slowed after 2016, with an upward trend emerging. After 2014, the incidence rate among females surpassed that of males, and the gender gap has continued to widen. In the Middle SDI region, the incidence rebounded after 1999 and increased rapidly, with females surpassing males in incidence rates, and the gender difference becoming more pronounced after 2016. In the Low-Middle SDI region, the incidence rose sharply starting in 1996, with a slowdown after 2008. In this region, incidence rates remained higher in males than females, with no significant change in the gender gap. Similarly, in the Low SDI region, the incidence increased rapidly from 1996 but slowed after 2002. Before 2015, there was no significant gender difference; however, after 2015, the incidence rate among females surpassed that of males, and the gender gap has continued to widen over time (Supplementary Figure 16).

When the joinpoint regression model was applied to different age groups, the trends in incidence rates varied. Globally, the 0–4 years and 5–9 years age groups showed declining incidence rates over the 30-year period. In contrast, the other three age groups exhibited upward trends, with the 15–19 years group demonstrating the most significant increase (AAPC = 0.29). In the High SDI region, all five age groups experienced declines in incidence rates, with the 5–9 years group showing the most substantial reduction (AAPC = −0.42). A similar pattern was observed in the High-Middle SDI region, where the 5–9 years group exhibited the most significant decline (AAPC = −0.58). In the Middle SDI region, the incidence rates declined for the 0–4 years and 5–9 years age groups, while the remaining three groups showed upward trends, with the most significant increase observed in the 15–19 years group (AAPC = 0.36). In both the Low-Middle SDI and Low SDI regions, incidence rates increased across all five age groups, with the 15–19 years group exhibiting the most pronounced rise (AAPC = 0.71 and 0.32, respectively) (Supplementary Figures 1–10).

### 3.3 Age, period, and cohort effects on pancreatitis, 1992–2021

In the global incidence of pancreatitis, the 0–4 years age group exhibited the lowest risk, with incidence rates progressively increasing with age. Females were at higher risk than males, a trend observed across most SDI regions. However, both globally and in the Middle SDI region, the incidence showed a positive increase after the 5–9 years age group. In the Low-Middle SDI and Low SDI regions, incidence increased across all age groups. Notably, in the High-Middle SDI region, the highest risk was observed in the 0–4 years age group, a pattern contrary to that seen in other SDI regions.

In terms of age-related effects, both globally and across all five SDI regions, pancreatitis incidence increased with age, with no significant gender differences observed within age groups.

For period effects, the overall incidence of pancreatitis among individuals aged 0–24 years increased over time. Notably, the gender disparity in pancreatitis incidence became more pronounced after the 2012–2016 period, with females showing a higher incidence risk than males. In five SDI regions, the risk decreased over time in the High SDI and High-Middle SDI regions, the other three SDI regions experienced a general increase in incidence risk throughout the 30-year period.

Regarding cohort effects, the global risk of pancreatitis increased in birth cohorts from 1997 to 2006, followed by a decline in subsequent cohorts. In the High SDI and High-Middle SDI regions, the cohort effect showed a declining trend, with younger birth cohorts exhibiting lower risks. Conversely, the Low, Low-Middle, and Middle SDI regions displayed an upward trend in incidence risk among younger birth cohorts, with the Middle SDI region closely aligning with the global trend.

In terms of DALYs, our analysis revealed a decreasing trend across all age groups, both globally and within the five SDI regions. The most significant decline was observed in the 0–4 years age group, with the rate of decline progressively slowing with increasing age.

In the context of age effects, DALYs rates increased with age globally and across all SDI regions, with the most marked rise occurring after the 10–14 years age group. Males consistently exhibited higher DALYs rates than females across all age groups.

For the period effect, the relative risk of DALYs for pancreatitis decreased over time, both globally and across the SDI regions, with the most substantial reduction observed in the High-Middle SDI region. In these regions, males maintained a consistently higher relative risk compared to females throughout the period analyzed.

Regarding the cohort effect, the relative risk of pancreatitis-related DALYs declined as birth cohorts became younger over the 30-year period, both globally and across the SDI regions. The most pronounced reduction was noted in the Middle SDI region. Across genders, males generally exhibited slightly higher relative risks than females.

**Figure 4:**
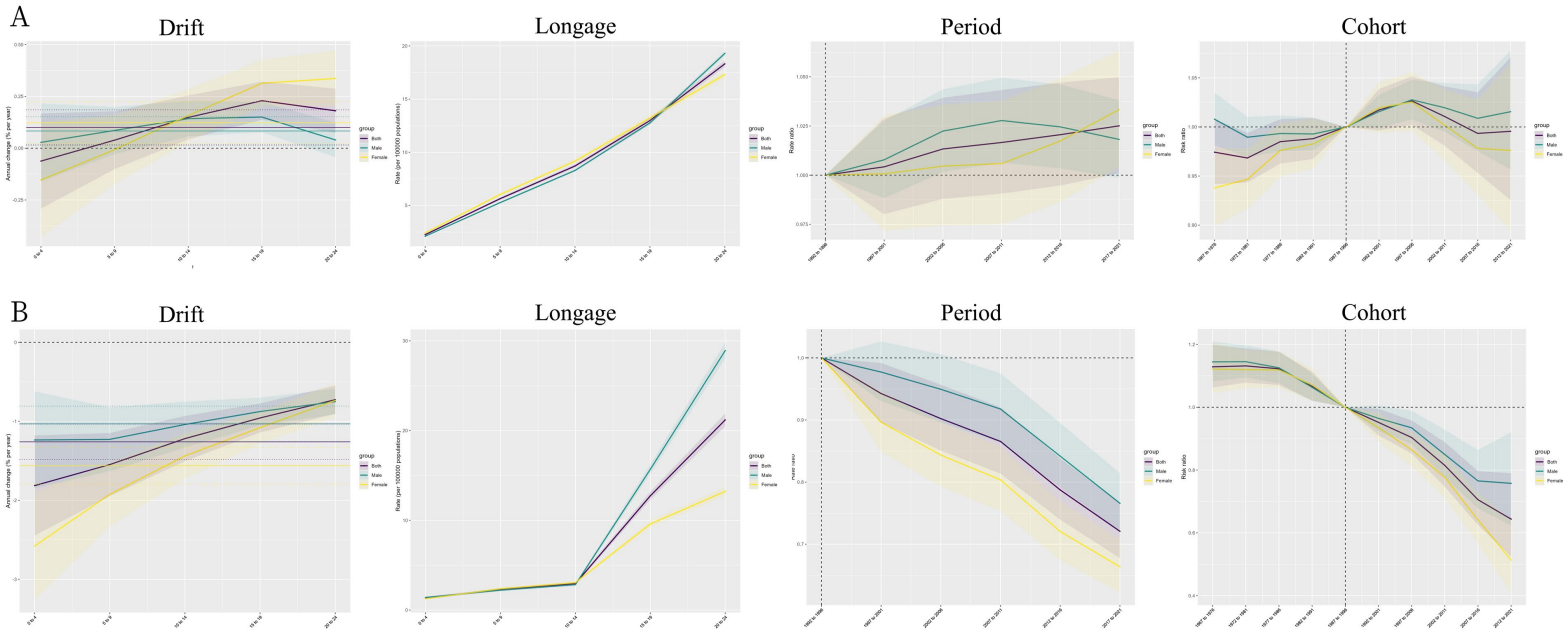
Age-period-cohort modeling of pancreatitis incidence and Disability-adjusted life-years (DALYs) in individuals aged 0–24 years globally. **(A)** Age-period-cohort modeling of pancreatitis incidence in individuals aged 0–24 years globally; **(B)** age-period-cohort modeling of pancreatitis disability-adjusted life-years (DALYs) in individuals aged 0–24 years globally.

Age-period-cohort modeling of global morbidity and disability-adjusted life years is shown in Figure 4. Age-period-cohort modeling of morbidity and disability-adjusted life years for the five SDI regions is shown in Supplementary Figures 11–15.

### 3.4 Global pancreatitis incidence and DALYs forecast for 0–24 years to 2040

**Figure 5:**
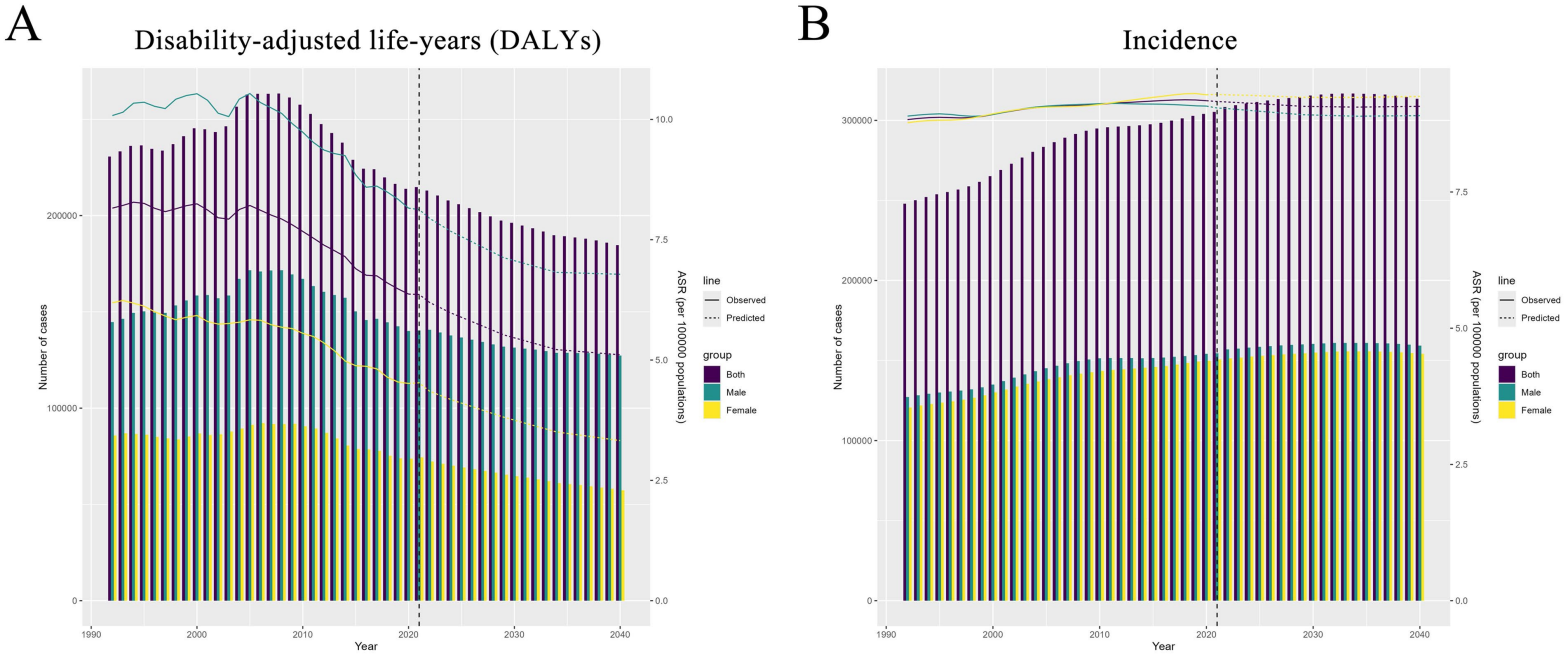
Norpred model prediction of pancreatitis incidence and disability-adjusted life-years (DALYs) in global populations aged 0–24 years. **(A)** Norpred model prediction of pancreatitis disability-adjusted life-years (DALYs) in global populations aged 0–24 years; **(B)** Norpred model prediction of pancreatitis incidence in global populations aged 0–24 years.

By 2040, the global number of pancreatitis cases among individuals aged 0–24 years is projected to reach 313,567, with an age-standardized incidence rate (ASIR) of 9.07 per 100,000 population. The projected global DALYs are estimated to total 184,691, with an ASR of 5.11. Eastern Europe is expected to maintain the highest incidence rate, with an ASR of 22.52, while South Asia is forecasted to have the largest number of cases, reaching 112,608. The projected trend for DALYs mirrors the incidence pattern, with Eastern Europe predicted to have the highest ASR of DALYs at 13.39, and South Asia anticipated to experience the highest total DALYs, amounting to 61,751 (Figure 5 and Supplementary material 5).

Forecasts for incidence across all age groups suggest that females aged 0–14 years will continue to exhibit higher incidence rates than males. In the 15–19 years age group, the number of cases among females is projected to become nearly equal to that of males, potentially surpassing it toward the latter part of the forecast period. However, for the 20–24 years age group, males are expected to have a significantly higher incidence rate and case count than females (Supplementary material 6).

The DALYs forecast indicates a decreasing trend across all age groups, although male DALYs will remain consistently higher than those of females. In particular, the gap in DALYs between males and females in the 15–24 years age group is expected to widen significantly over time (Supplementary Figure 16).

## 4 Discussion

Pancreatitis predominantly affects middle-aged and older adult populations (13, 14), the incidence in younger populations is considerably lower. For example, Xiao et al. (15) reported an ASR of 33.74 per 100,000 for acute pancreatitis and 9.62 per 100,000 for chronic pancreatitis among older populations. In contrast, the highest incidence among young people in 2021 in our study was observed in Eastern Europe, with an ASR of 23.74 (95% CI: 14.59–36.54). This result is consistent with the findings of Roberts et al. (16). Given the scarcity of high-quality epidemiological studies on pancreatitis in certain regions, particularly Europe, the insights provided by this study are valuable in bridging existing knowledge gaps and informing public health strategies.

Russia reported the highest incidence rate of pancreatitis in 2021, with an age-standardized incidence rate of 24.7 (95% CI: 15.13–38.23). A notable gender disparity was observed, with the incidence rate being higher among males (27.11, 95% CI: 16.13–43.83) than females (22.18, 95% CI: 13.75–32.7). Alcohol consumption is a well-established risk factor for both acute and chronic pancreatitis (17), and Russia is recognized for its high levels of alcohol consumption, particularly among males (18). Moreover, the prevalence of binge drinking among adolescents in Russia has been increasing in recent years (19), aligning with the findings of this study. These behavioral patterns likely contribute to the elevated incidence rates of pancreatitis, especially among younger males, underscoring the importance of targeted public health interventions to reduce alcohol consumption and mitigate the disease burden.

South Asia and India were identified as the region and country, respectively, with the highest number of pancreatitis cases. Over the past 30 years, the incidence of pancreatitis among young people has shown an overall upward trend globally, with South Asia experiencing the most significant increase (AAPC = 0.91, 95% CI: 0.87–0.96). As India accounts for a large portion of the South Asian population, it has also shown a marked increase in incidence, with an AAPC of 1.07 (95% CI: 1.03–1.11). Although the etiology of pancreatitis is complex, factors such as high population density and relatively poor sanitation likely contribute to the disease burden in South Asia. In India, ascariasis is a significant cause of biliary and pancreatic diseases, with acute pancreatitis due to ascariasis accounting for a substantial proportion of cases (20–22). This finding highlights the need for local policymakers to implement targeted policies aimed at protecting children and adolescents from preventable causes of pancreatitis. Improving sanitation, controlling parasitic infections, and raising awareness about early diagnosis and management are essential steps to reduce the burden of the disease in the region.

The trends in DALYs at the regional and national levels closely mirrored those observed for incidence rates, with one notable exception: Kiribati exhibited the highest age-standardized DALYs rate. DALYs are strongly influenced by the quality of healthcare, and both India and Kiribati face significant challenges in this area (23). In contrast, the high DALYs burden observed in Russia, despite being a high-SDI country, is closely tied to excessive alcohol consumption within its population (18). This highlights the multifaceted nature of pancreatitis burden, where factors such as healthcare infrastructure, environmental conditions, and lifestyle behaviors interact to shape regional and national outcomes. Addressing these burdens will require region-specific strategies, including improving healthcare access and quality, reducing alcohol consumption, and promoting public health initiatives targeted at children and adolescents.

Chile exhibited the fastest increase in pancreatitis incidence, with an AAPC of 1.7 (95% CI: 1.66–1.74). Although pancreatitis imposes a significant burden on Chile’s young population, the current lack of high-quality national-level data limits a comprehensive evaluation of this trend (24). This study provides valuable insights that can serve as a useful reference for future research and policy planning. In contrast, Eastern Asia, particularly China, has shown the most significant declines in pancreatitis incidence among young people over the past 30 years. Alcohol-related pancreatitis, which is more prevalent in regions such as Europe and North America (25, 26), is not a primary cause of the disease in China. Furthermore, alcohol consumption among Chinese adolescents remains lower than in Europe and North America (27). However, due to China’s large population size, the overall burden of pancreatitis remains considerable despite the declining incidence trend.

Advancements in diagnostic and imaging technologies have enhanced the early detection of pancreatic diseases (28). Early diagnosis and timely treatment are critical for managing pancreatitis and reducing its incidence (29, 30), especially in pediatric cases (31). Consistent with these developments, our APC model found that the incidence of pediatric pancreatitis has declined more significantly than in other age groups over the past 30 years. Progress in treatments—such as endoscopic therapies, management of pancreatic complications, and care for severe pancreatitis—has also contributed to reducing adverse outcomes and improving prognosis (32–34). Our findings reflect these improvements, with the APC model showing a global decline in pancreatitis-related DALYs across the five SDI regions. The most pronounced improvements were observed in the 0–4 years age group, suggesting that advances in pediatric care have been particularly impactful. However, significant disparities persist between high-income and low-income countries, underscoring the need for increased public health investment in underdeveloped regions. Allocating resources toward improving diagnostic capacity, treatment accessibility, and healthcare infrastructure in low-income settings is essential to ensure equitable health outcomes and further reduce the global burden of pancreatitis.

Although the age-standardized rate of DALYs among individuals aged 0–24 years has shown a general decline over the past 30 years, males consistently exhibit higher DALYs than females across most regions. A retrospective study on acute pancreatitis reported that males tend to experience worse prognoses than females (35). While that study primarily focused on older populations, our findings reveal a similar trend among children and adolescents, highlighting the need to prioritize disease management in male patients. The burden of pancreatitis varies significantly across different age groups. In our study, the incidence of pancreatitis among children (0–9 years) demonstrated an overall decline globally over the past 30 years, while the incidence among adolescents (10–24 years) increased. However, this pattern diverges across regions. In relatively developed regions, the incidence declined across all age groups, likely reflecting improved healthcare access, early detection, and preventive measures. In contrast, the opposite trend was observed in some underdeveloped regions, where limited healthcare resources, delayed diagnoses, and persistent risk factors have contributed to a rising incidence across both children and adolescents.

The etiology of pediatric pancreatitis is more diverse compared to that in adults (4). Genetic factors play a relatively larger role in acute recurrent pancreatitis and chronic pancreatitis in children (36). Consequently, in less developed regions, it is essential to consider not only environmental factors, trauma, and metabolic disorders but also potential genetic influences during pregnancy that may increase fetal susceptibility to pancreatitis. Further research is required to better understand gene–environment interactions contributing to the development of pancreatitis in these settings. Our age-period-cohort (APC) model reveals that the incidence and DALYs of pancreatitis increase with age, particularly after the age of 10, as adolescents become more exposed to social environments. Known risk factors such as smoking and alcohol consumption are of particular concern in this age group (37). Despite public health efforts, the burden of adolescent smoking and drinking remains high (38), underscoring the need for stricter public health policies and interventions. Targeted initiatives focused on reducing substance use in adolescents are essential to mitigate these risks and curb the burden of pancreatitis.

Previous studies suggested that the incidence of pancreatitis was similar between males and females (39), however, we observed that, since 2016, the incidence rate of pancreatitis among females has surpassed that of males, with the gap continuing to widen. Our forecasting model predicts that the incidence rate among females aged 0–24 years will remain higher than that of males, particularly in the 0–19 years age group, where the ASR for females is consistently higher. Endocrine hormones, particularly oestrogen, may contribute to pancreatic dysfunction in female patients, thereby exacerbating the risk of pancreatitis (40), especially in adolescent females. Additionally, studies have indicated that certain types of pancreatitis in children, such as hereditary pancreatitis and autoimmune pancreatitis, present a higher incidence and earlier onset in females (31, 41). These highlighting the importance of monitoring the emerging disease burden among females. This shift underscores the need for further research into gender differences to better understand the underlying factors driving these trends. Such insights will be essential to develop targeted public health policies and interventions that effectively address the distinct needs of female patients, particularly during childhood and adolescence.

Our predictive model indicates that by 2040, the burden of pancreatitis among children and adolescents will remain significant, with a steady increase in incidence, particularly within the 15–24 age group. To address this, stronger policy support is essential. Tobacco and alcohol consumption have been identified as major risk factors for pancreatitis, especially among adolescents. Strengthening regulations and increasing taxation on these products can effectively reduce consumption. Evidence also suggests that tobacco control policies and alcohol restrictions have a significant impact on reducing pancreatitis-related diseases (42). In addition, public health campaigns and education initiatives are crucial. Health education programs that raise awareness among adolescents about the adverse effects of smoking and alcohol consumption on pancreatic health can help reduce high-risk behaviors. Furthermore, screening adolescents with a family history or established smoking and drinking behaviors for early detection of pancreatic issues is necessary to mitigate future disease burden.

This study has several limitations. First, due to the constraints of the GBD data, we were unable to perform more detailed classification analyses of acute pancreatitis (AP) and chronic pancreatitis (CP). Second, important risk factors for pediatric and adolescent pancreatitis—such as congenital biliary and pancreatic diseases, gallstones, and hypercholesterolemia—could not be fully analyzed, as the current GBD database lacks subtype data categorized by these underlying causes. To address these gaps, continuous efforts are needed to integrate new data sources into the GBD database, allowing for more comprehensive analyses of pancreatitis subtypes and their risk factors. Future research should focus on expanding data granularity to improve the understanding of disease etiology and better inform targeted public health strategies.

## 5 Conclusion

In conclusion, this study provides a comprehensive overview of the changes in the burden of pancreatitis among children and adolescents globally and across regions over the past 30 years, using incidence, DALYs, and related ASR indicators. From 1992 to 2021, the burden of pancreatitis in this population has steadily increased, with notable variations across age groups and genders. Although overall disease outcomes have improved compared to previous years, targeted public health interventions are essential to mitigate the burden of the disease, particularly by addressing modifiable risk factors such as alcohol consumption among young people. A concerted effort to promote early detection, prevention, and appropriate management will be critical in reducing the long-term impact of pancreatitis on this vulnerable population.

## Data Availability

The original contributions presented in the study are included in the article/Supplementary material, further inquiries can be directed to the corresponding author.

## Footnotes

^1^ https://vizhub.healthdata.org/gbd-results/
