# Supplementary material for: Global, regional, and national burdens of pancreatitis in children and adolescents aged 0–24 years from 1992 to 2021: a trend analysis based on the global burden of disease study 2021
Source: Front Public Health. 2025 Jun 26;13:1527569. doi: 10.3389/fpubh.2025.1527569 (PMC12240931; doi:10.3389/fpubh.2025.1527569)

**Figure 1:**Global, regional incidence and Disability-adjusted life-years (DALYs) of pancreatitis in various age subgroups.

Figure (1A):Disability-adjusted life-years (DALYs) rate for pancreatitis in various age subgroups in Global and 21 GBD regions; (1B):Incidence for pancreatitis in various age subgroups in Global and 21 GBD regions; (1C):Disability-adjusted life-years (DALYs) and incidence cases for pancreatitis in 5 SDI regions

**Figure 2:**Age-standardized incidence and Disability-adjusted life-years (DALYs) rates for pancreatitis among individuals aged 0-24 years across 204 countries worldwide in 1992 and 2021.

Figure (2A)Age-standardized Disability-adjusted life-years (DALYs) rate for pancreatitis among individuals aged 0-24 years across 204 countries worldwide in 1992; (2B)Age-standardized Disability-adjusted life-years (DALYs) rate for pancreatitis among individuals aged 0-24 years across 204 countries worldwide in 2021; (2C)Age-standardized incidence rate for pancreatitis among individuals aged 0-24 years across 204 countries worldwide in 1992; (2D)Age-standardized incidence rate for pancreatitis among individuals aged 0-24 years across 204 countries worldwide in 2021.

**Figure 3:**Joinpoint modeling of the age-standardized incidence and Disability-adjusted life-years (DALYs) rates of pancreatitis in individuals aged 0-24 years globally.

Figure (3A):Joinpoint modeling of the age-standardized Disability-adjusted life-years (DALYs) rate of pancreatitis in individuals aged 0-24 years globally; (3B)Joinpoint modeling of the age-standardized incidence rate of pancreatitis in individuals aged 0-24 years globally.

**Figure 4:**Age-period-cohort modeling of pancreatitis incidence and Disability-adjusted life-years (DALYs) in individuals aged 0-24 years globally.

Figure (4A):Age-period-cohort modeling of pancreatitis incidence in individuals aged 0-24 years globally; (4B)Age-period-cohort modeling of pancreatitis Disability-adjusted life-years (DALYs) in individuals aged 0-24 years globally.

**Figure 5:**Norpred model prediction of pancreatitis incidence and Disability-adjusted life-years (DALYs) in global populations aged 0-24 years.

Figure 5(A):Norpred model prediction of pancreatitis Disability-adjusted life-years (DALYs) in global populations aged 0-24 years;(B):Norpred model prediction of pancreatitis incidence in global populations aged 0-24 years.

**Supplementary Figures 1–10, 16:**Joinpoint modeling of the incidence and Disability-adjusted life-years (DALYs) rates of pancreatitis in age subgroups globally and in 5 SDI regions.

Supplementary Figures 1:Joinpoint modeling of the Disability-adjusted life-years (DALYs) rate of pancreatitis in age 0-4 years globally and in 5 SDI regions.


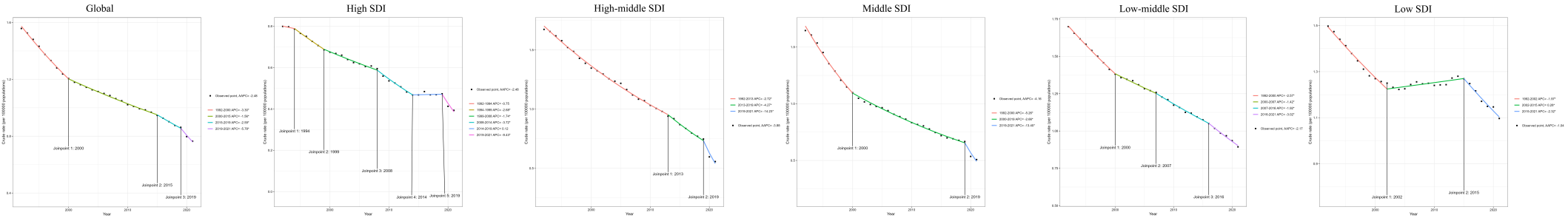


Supplementary Figures 2:Joinpoint modeling of the Disability-adjusted life-years (DALYs) rate of pancreatitis in age 5-9 years globally and in 5 SDI regions.


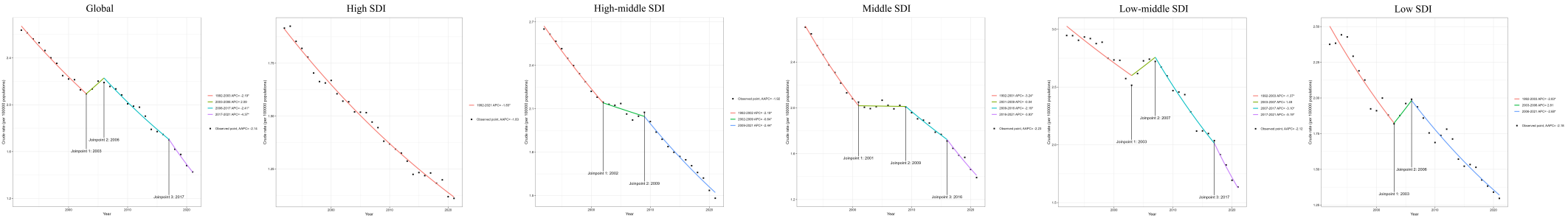


Supplementary Figures 3:Joinpoint modeling of the Disability-adjusted life-years (DALYs) rate of pancreatitis in age 10-14 years globally and in 5 SDI regions.


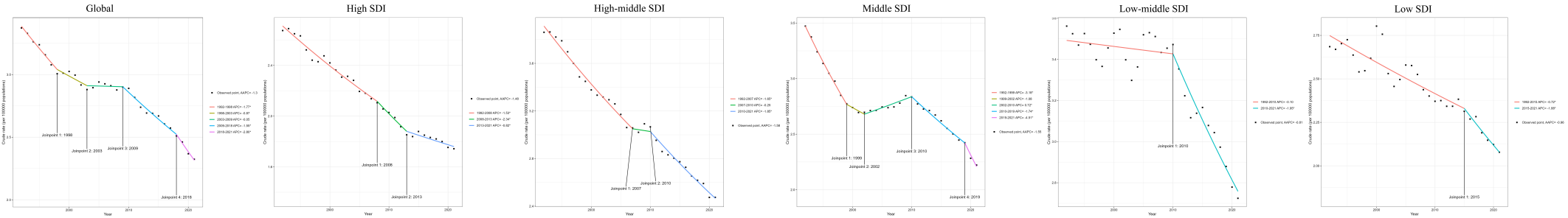


Supplementary Figures 4:Joinpoint modeling of the Disability-adjusted life-years (DALYs) rate of pancreatitis in age 15-19 years globally and in 5 SDI regions.


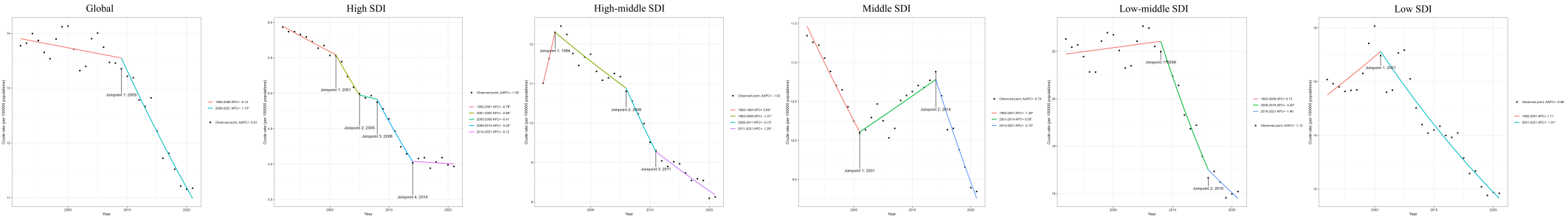


Supplementary Figures 5:Joinpoint modeling of the Disability-adjusted life-years (DALYs) rate of pancreatitis in age 20-24 years globally and in 5 SDI regions.


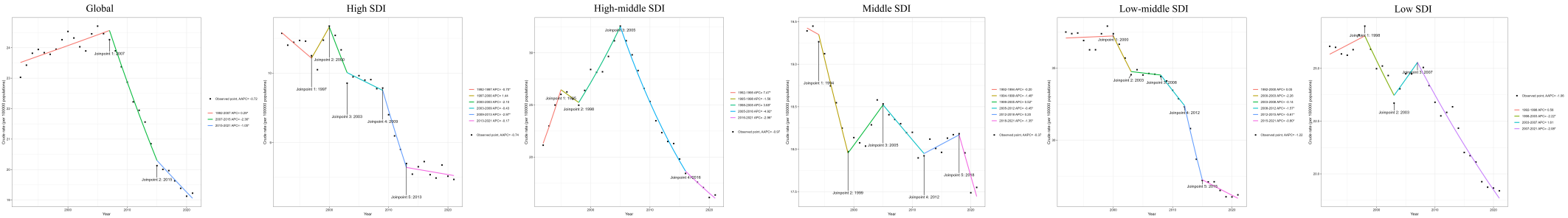


Supplementary Figures 6:Joinpoint modeling of the incidence rate of pancreatitis in age 0-4 years globally and in 5 SDI regions.


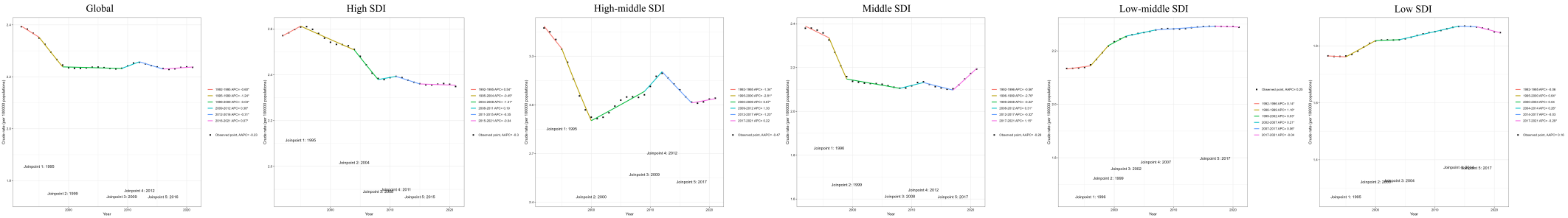


Supplementary Figures 7:Joinpoint modeling of the incidence rate of pancreatitis in age 5-9 years globally and in 5 SDI regions.


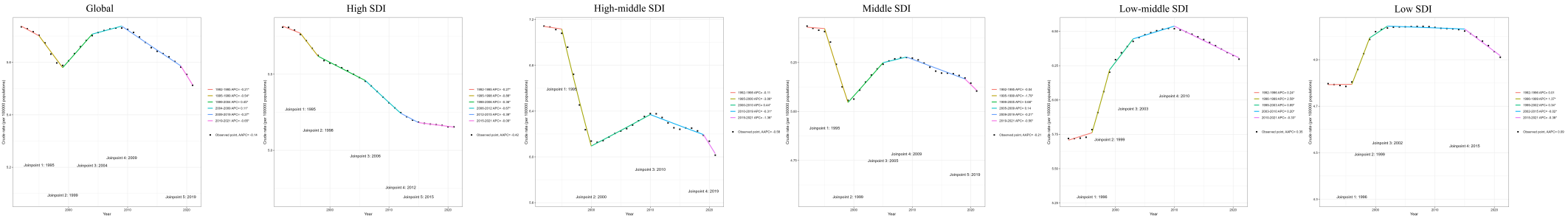


Supplementary Figures 8:Joinpoint modeling of the incidence rate of pancreatitis in age 10-14 years globally and in 5 SDI regions.


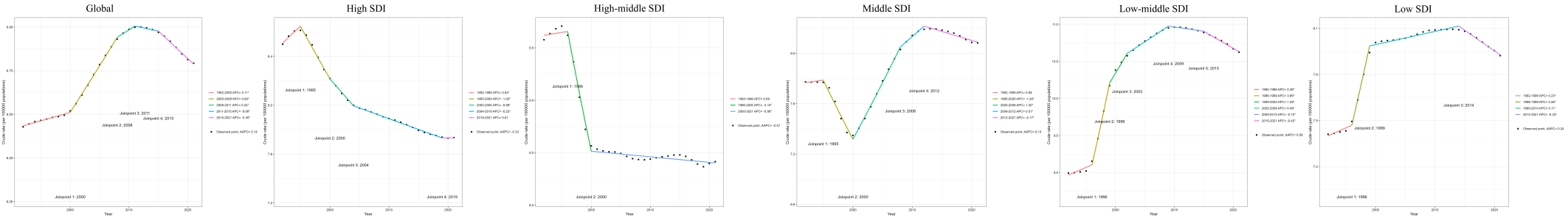


Supplementary Figures 9:Joinpoint modeling of the incidence rate of pancreatitis in age 15-19 years globally and in 5 SDI regions.


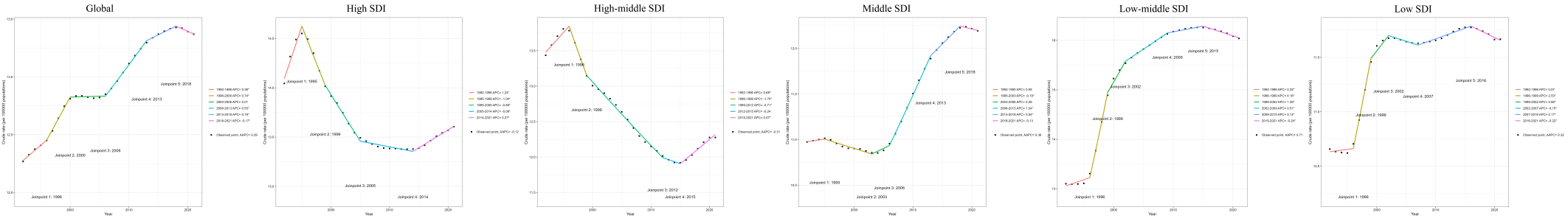


Supplementary Figures 10:Joinpoint modeling of the incidence rate of pancreatitis in age 20-24 years globally and in 5 SDI regions.


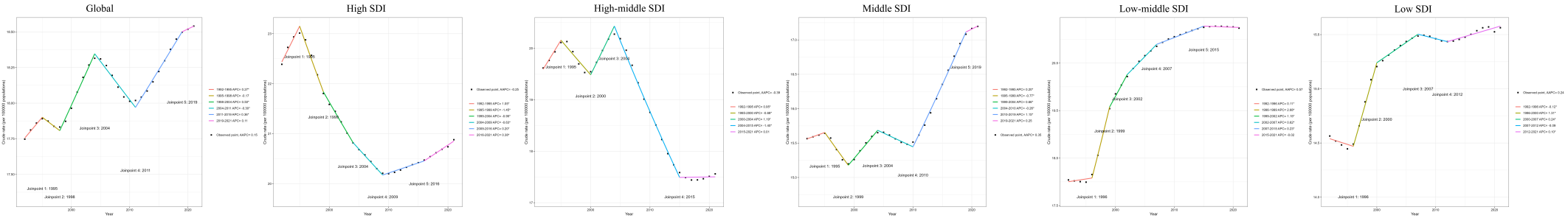


Supplementary Figures 16:Joinpoint modeling of the age-standardized incidence and Disability-adjusted life-years (DALYs) rates of pancreatitis in individuals aged 0-24 years in 5 SDI regions.

Supplementary Figures 16(A):Joinpoint modeling of the age-standardized Disability-adjusted life-years (DALYs) of pancreatitis in individuals aged 0-24 years in 5 SDI regions; (B)Joinpoint modeling of the age-standardized incidence of pancreatitis in individuals aged 0-24 years in 5 SDI regions.


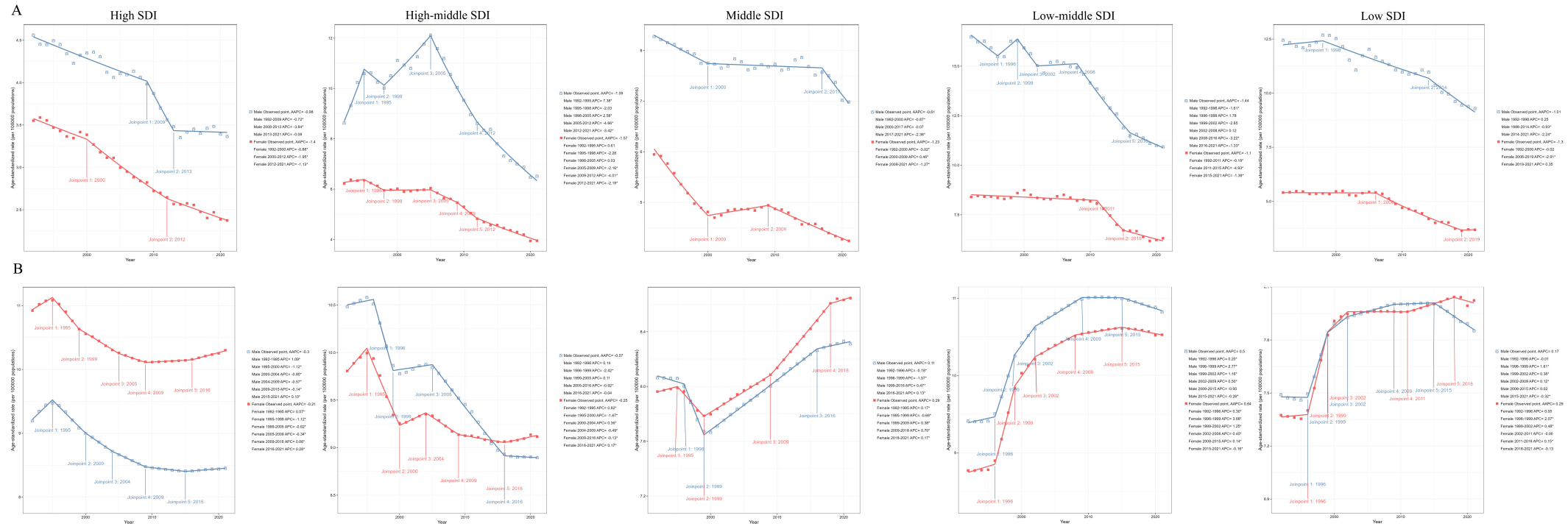


**Supplementary Figures 11–15:**Age-period-cohort modeling of pancreatitis incidence and DALYs in individuals aged 0-24 years in 5 SDI regions.

Supplementary Figures 11(A):Age-period-cohort modeling of pancreatitis Disability-adjusted life-years (DALYs) in individuals aged 0-24 years in high SDI region; (B)Age-period-cohort modeling of pancreatitis incidence in individuals aged 0-24 years in high SDI region.


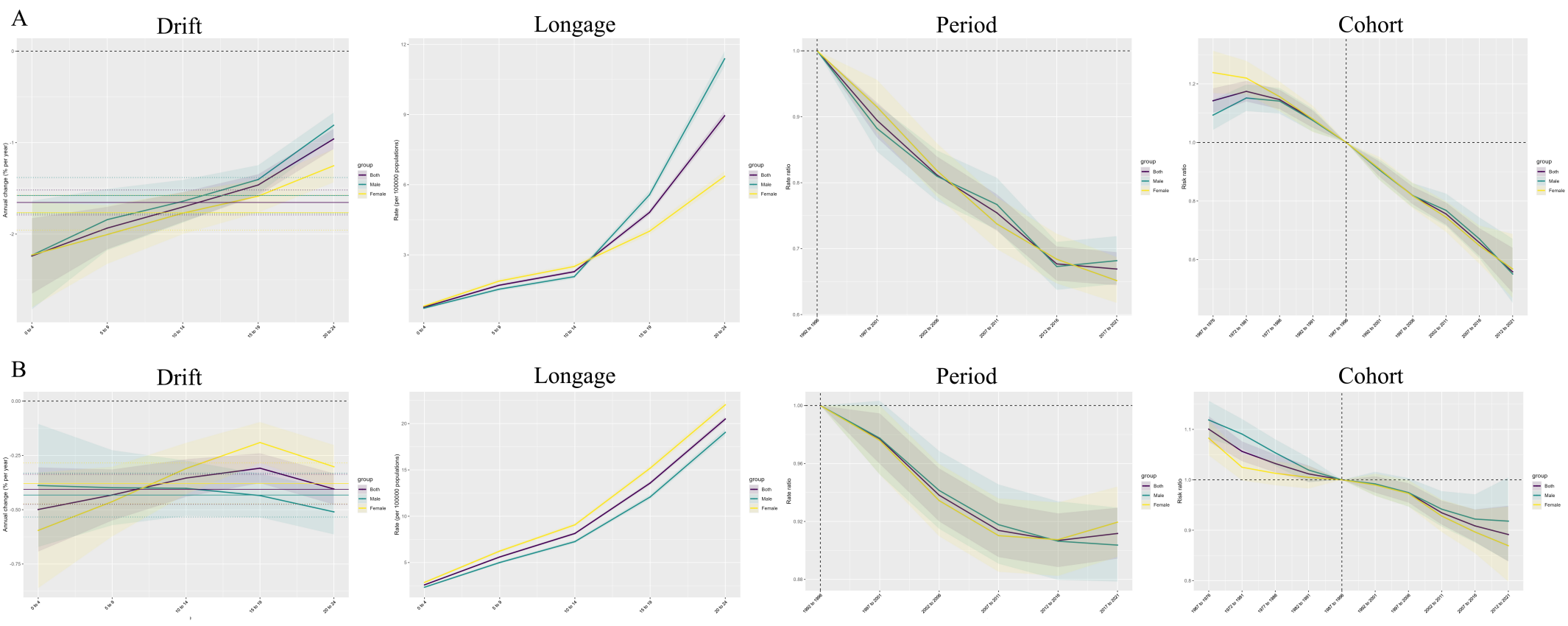


Supplementary Figures 12(A):Age-period-cohort modeling of pancreatitis Disability-adjusted life-years (DALYs) in individuals aged 0-24 years in high-middle SDI region; (B)Age-period-cohort modeling of pancreatitis incidence in individuals aged 0-24 years in high-middle SDI region.


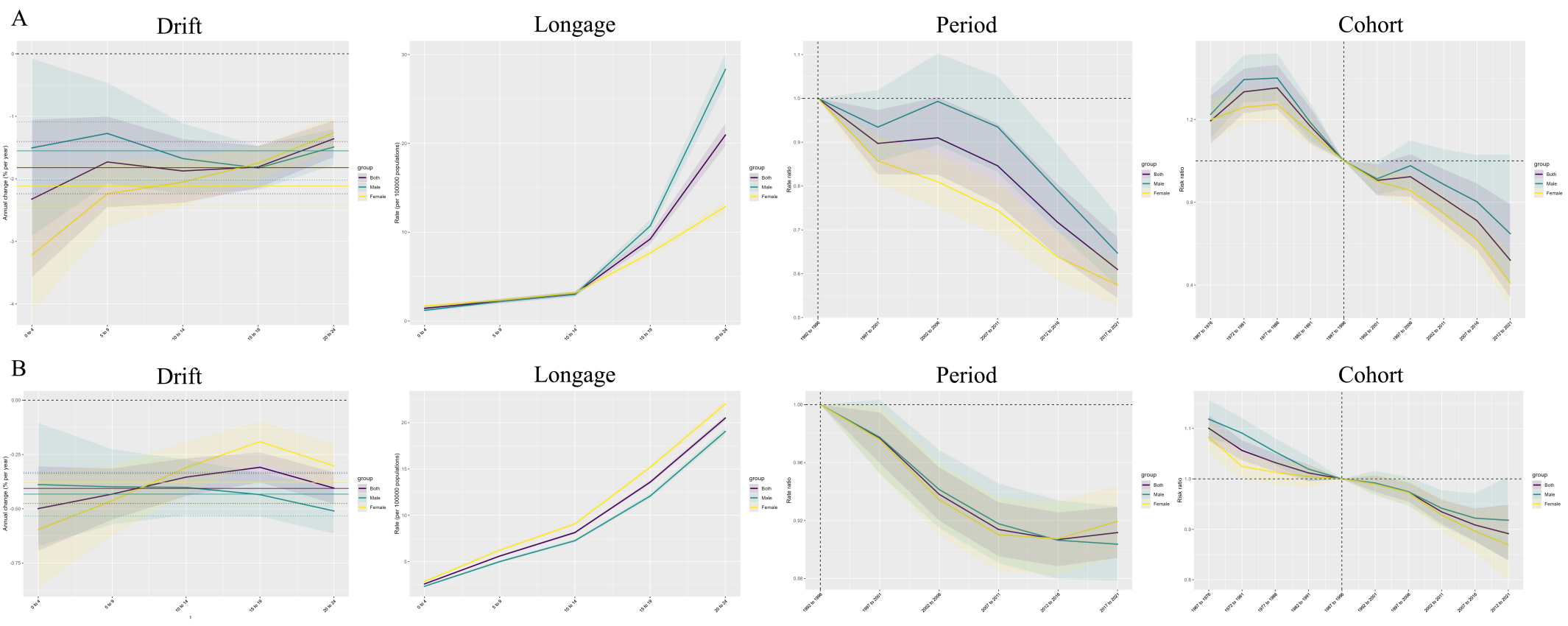


Supplementary Figures 13(A):Age-period-cohort modeling of pancreatitis Disability-adjusted life-years (DALYs) in individuals aged 0-24 years in middle SDI region; (B)Age-period-cohort modeling of pancreatitis incidence in individuals aged 0-24 years in middle SDI region.


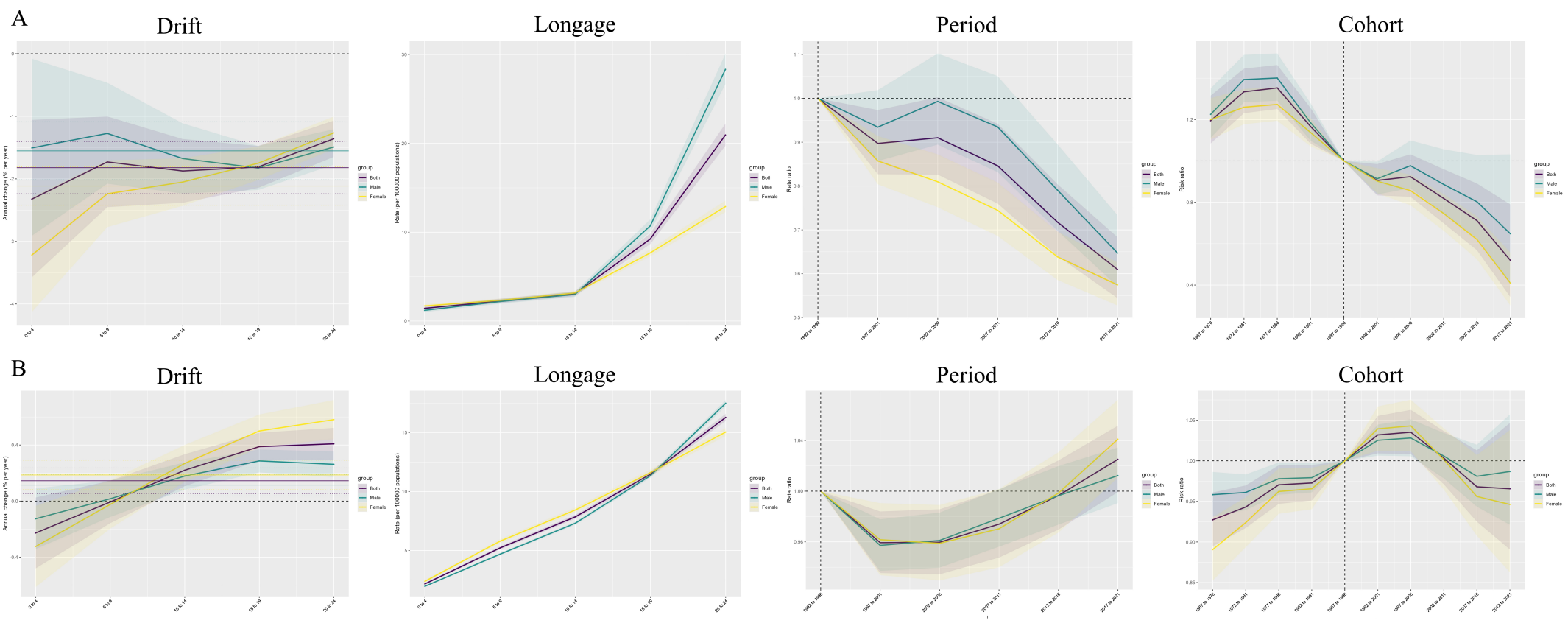


Supplementary Figures 14(A):Age-period-cohort modeling of pancreatitis Disability-adjusted life-years (DALYs) in individuals aged 0-24 years in low-middle SDI region; (B)Age-period-cohort modeling of pancreatitis incidence in individuals aged 0-24 years in low-middle SDI region.


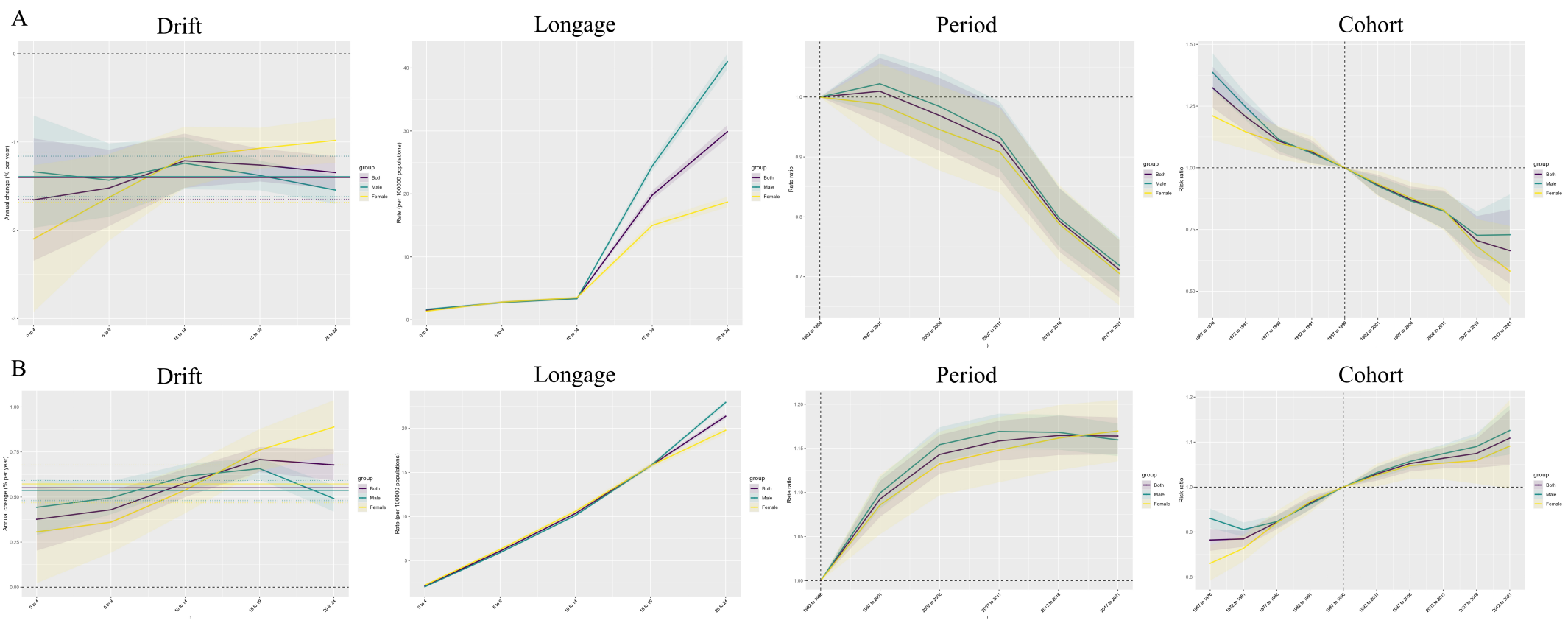


Supplementary Figures 15(A):Age-period-cohort modeling of pancreatitis Disability-adjusted life-years (DALYs) in individuals aged 0-24 years in low SDI region; (B)Age-period-cohort modeling of pancreatitis incidence in individuals aged 0-24 years in low SDI region.


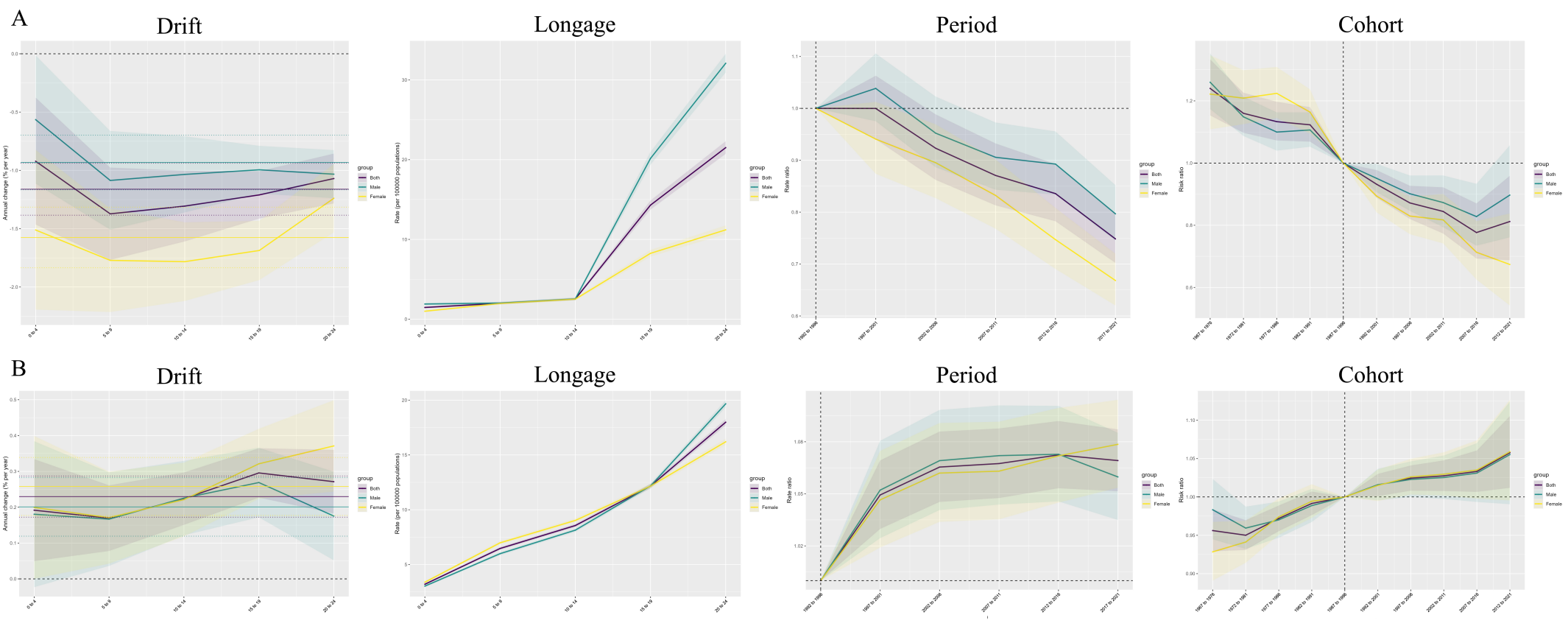


**Supplementary Figures 17**:Norpred model prediction of pancreatitis incidence and Disability-adjusted life-years (DALYs) in global populations in different age group.

Supplementary Figures 17(A):Norpred model prediction of pancreatitis Disability-adjusted life-years (DALYs) in global populations in different age group; (B):Norpred model prediction of pancreatitis incidence in global populations in different age group.


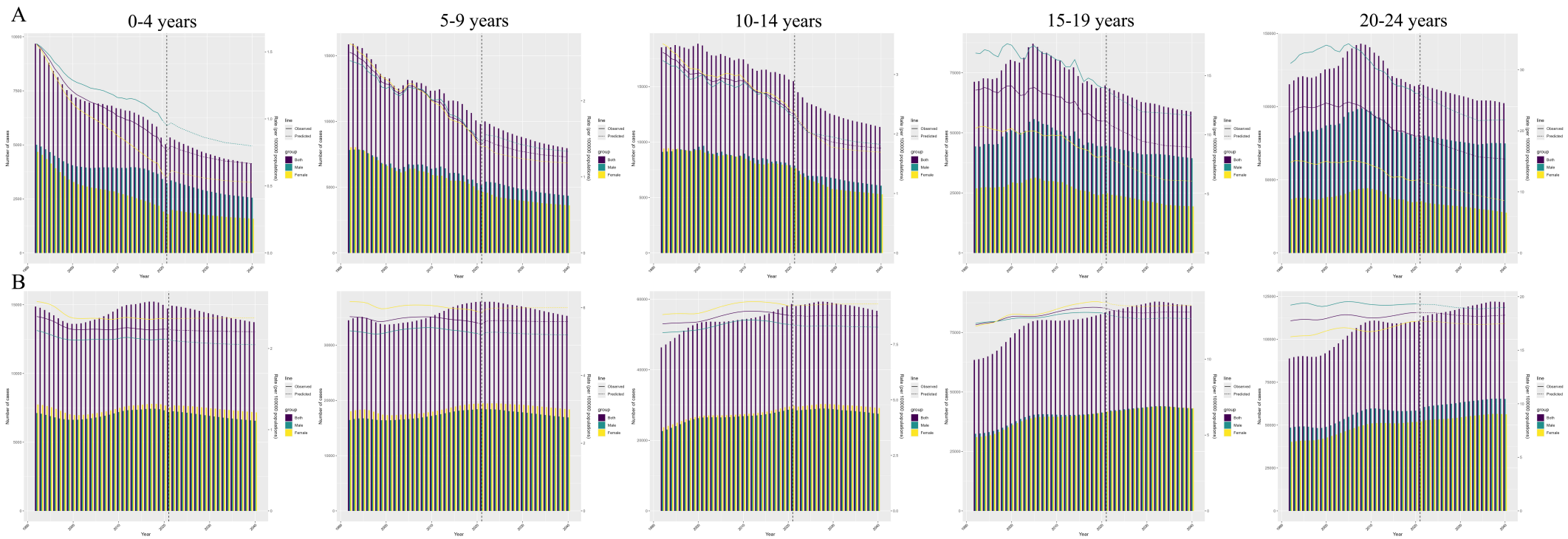

Supplement: Supplementary file 7 [file Data_Sheet_7.docx]
