# Supplementary material for: Global, regional, and national burdens of pancreatitis in children and adolescents aged 0–24 years from 1992 to 2021: a trend analysis based on the global burden of disease study 2021
Source: Front Public Health. 2025 Jun 26;13:1527569. doi: 10.3389/fpubh.2025.1527569 (PMC12240931; doi:10.3389/fpubh.2025.1527569)
Supplement: Supplementary file 8 [file Data_Sheet_8.docx]

**Abbreviations**

| **Abbreviation** | **Definition** |
| --- | --- |
| GBD | Global Burden of Disease |
| SDI | Socio-Demographic Index |
| WHO | World Health Organization |
| APC | Annual Percentage Change |
| AAPC | Average Annual Percentage Change |
| APC | Age-Period-Cohort |
| ASR | Age-Standardized Rate |
| YLL | Years of Life Lost due to  premature death |
| YLD | Years Lost due to Disability |
| DALYs | Disability-Adjusted Life Years |
| ASIR | Age-Standardized Incidence Rate |
| ASDR | Age-Standardized DALYs Rate |

Note 1:Age-Standardized Rate=$\frac{\sum_{i=1}^{A} aiwi}{\sum_{i=1}^{A} wi}$×100,000^1^.

Note 2:DALYs refer to the total healthy life years lost from morbidity to death, including years of life lost due to premature death (YLL) and years lost due to disability (YLD). The calculation formula of YLL is YLL =N*L,where N represents the number of deaths and L represents the standard life expectancy at the age of death in years. The calculation formula of YLD is YLD= I*DW*L,where I is the number of incident cases, DW is the disability weight, and L is the average duration of disability years. Disability weight represents the severity of health loss associated with a single health condition^2^.

**References**

1. Hankey BF, Ries LA, Kosary CL, et al. Partitioning linear trends in age-adjusted rates. *Cancer Causes Control* 2000; **11**(1): 31-5.

2. Salomon JA, Haagsma JA, Davis A, et al. Disability weights for the Global Burden of Disease 2013 study. *Lancet Glob Health* 2015; **3**(11): e712-23.
